# Supplementary material for: A protective and broadly binding antibody class engages the influenza virus hemagglutinin head at its stem interface
Source: mBio. 2025 May 20;16(6):e00892-25. doi: 10.1128/mbio.00892-25 (PMC12153317; doi:10.1128/mbio.00892-25)
Supplement: Supplemental material — Table S1 and Figures S1 to S8. [file mbio.00892-25-s0001.docx]

**Supporting Figures:**

**Supporting Table 1: Data collection and refinement statistics.**

**Figure S1: S8V1-157 Fab tightly binds monomeric HA head.** A. BLI traces showing the association of S8V1-157 Fab with H3/X-31 head-loaded BLI sensors. Colored traces denote empirical binding data for different concentrations of Fab; black traces denote the calculated curves of best global fit for a 1:1 binding model. B. BLI traces showing the association of S5V2-29 Fab with H3/X-31 head-loaded BLI sensors. C. BLI traces showing the association of S8V1-157 Fab or S5V2-29 Fab with H3/X-31 head- or H3/X-31 FLSE-loaded BLI sensors. Both fail to engage a full-length HA ectodomain. D. BLI traces showing the association of CR8020 Fab with H3/X-31 FLSE-loaded BLI sensors. Vertical black lines in panels A-D denote the start of the association phase (10 s) and dissociation phase (130 s). In all traces, protein binding to the loaded sensor was measured as wavelength shift (Δλ) in nm. E. Summary of BLI binding data for HAs that were bound by Fabs. F. S8V1-157 forms a stable complex with monomeric HA head. Equal molar ratios of S8V1-157 Fab and HA H3/X-31 head were co-concentrated, incubated for 1 hour at room temperature and analyzed using size exclusion chromatography and collected in 1ml fractions. Equal amounts of S8V1-157 Fab and H3/X-31 head were run as controls. Proteins from the indicated fractions were analyzed by SDS-PAGE and stained with Coomassie blue. Protein ladder weights (kDa) top to bottom are: 180, 130, 100, 70, 55, 40, 35, 25, 15 and 10.

**Figure S2: S8V1-157 Contacts conserved sites.** An alignment of HA head regions for HAs used in this study. S8V1-157 contacting residues in A/American black duck/New Brunswick/00464/2010(H4N6) are colored red. HAs not bound by S8V1-157 are in denoted in gray. Sites identical to the A/American black duck/New Brunswick/00464/2010(H4N6) sequence are indicated by a “.”. Amino acid numbering corresponds to standard H3N2 numbering. HAs bound by these antibodies were used to generate the conservation diagram in Figure 2.

**Figure S3: Surface hydrophobicity on the HA molecule.** Top: Surface hydrophobicity and the head-stem epitope are shown on the HA trimer of A/American black duck/New Brunswick/00464/2010(H4N6) (PDB: 5XL2) ^22^. Coloring is based on the PyMOL Color h script that utilizes a normalized consensus hydrophobicity scale^62^. Views and orientations match those in Figure 2. The head-stem epitope is circled in the far right panel. Bottom: An electron density 2Fo-Fc map for the HA-Fab interacting region contorted at sigma level 1.0

**Figure S4: ELISA titrations of antibodies on HA coated plates.** The broadly binding stem antibody FI6v3^48^ was used as a positive control and an influenza B specific head antibody, CR8071^59^, as a negative control for influenza A isolates. Data points represent the average of three technical replicates. The standard error of the mean is shown for each point. KDs were calculated from the curves fit to these data points.

**Figure S5: Head-stem epitope antibody binding to HA head-only constructs.** A. Structures of HA head used in these experiments the S8V1-157-HA head complex is shown for reference. A compact head^50^ , is shown for reference (PDB 7TRH). B. HA heads for HAs not bound or not expressed in Figure 3 were produced alongside a positive control A/California/07/2009(H1N1)(X-181) and a compact head version. Sequences of the HA head regions are aligned to the A/Aichi/02/1968(H3N2)(X-31) reference sequence and numbered by H3 convention. C. Dissociation constants from ELISA measurements of head-stem epitope antibodies to HA heads. Head interface antibody FluA-20^41^ was used as a positive control and influenza B head antibody CR8071^59^ as a negative control.

**Figure S6: ELISA titrations of antibodies on HA head coated plates and characterization of cell expressed HA.** A. Head interface antibody FluA-20^41^ was used as a positive control and influenza B head antibody CR8071^59^ as a negative control. Data points represent the average of three technical replicates. The standard error of the mean is shown for each point. KDs were calculated from the curves fit to these data points. B. Lysates from 293F transfected with either HA from A/Aichi/02/1968(H3N2)(X31) or empty vector were subjected to western blotting under reducing and denaturing conditions with either anti-HA tag antibody, which recognizes an endogenous sequence in the H3 HA head (HA1), or anti-GAPDH antibody. Molecular weights of unprocessed HA0 and processed HA1 are indicated with open or closed arrowheads, respectively. C. 293F transfected with either HA from A/Aichi/02/1968(H3N2)(X31) or empty vector were stained with the indicated antibodies and analyzed by flow cytometry. Gates denoting cells bound by antibody are shown, with the percent of the total population indicated above.

**Figure S7: Functions of S8V1-157**. A. S8V1-157 is a non-neutralizing antibody. Neutralization IC50 values for S8V1-157, neutralizing antibody HC19^33^ (positive control) and SARS-CoV antibody CR3022^34^ (negative control).B. S8V1-157 bound to HA signals through FcγRIV. Results of an in vitro ADCC proxy assay. Mouse FcγRIV-expressing effector cells and HA-expressing target cells were cocultured in the presence of serially diluted recombinant mouse IgG2c Abs. FcγRIV activation was measured as luminescence output, as described in Materials and Methods. Error bars: mean ± S.D. of three technical replicates. Data shown are representative of two independent experiments with similar results. In these experiments SARS-CoV antibody CR3022^34^ was used as a negative control and FI6v3^48^ as a positive control.

**Figure S8: Antibody genetics of S8V1-157 competing antibodies.** Antibody names, gene usage and HCDR3 sequences for S8V1-157 competing antibodies.

**REFERENCE**:

62 Eisenberg, D., Schwarz, E., Komaromy, M. & Wall, R. Analysis of membrane and surface protein sequences with the hydrophobic moment plot. *J Mol Biol* **179**, 125-142 (1984). <https://doi.org:10.1016/0022-2836(84)90309-7>
